# AI-MDT: an automatic and intelligent multidisciplinary team consultations platform for lung cancer diagnosis

**DOI:** 10.1007/s00432-025-06413-5

**Published:** 2026-01-08

**Authors:** Yunyou Liu, Fei Wang, Peng Wang, Zhen Zhou, Hongqian Wang, Jingyao Li, Yang Qiu, Haidong Wang, Siwei Miao

**Affiliations:** 1 Department of Clinical Laboratory, Southwest Hospital, Third Military Medical University (Army Medical University), Chongqing, 400038 China; 2 Centre for Medical Big Data and Artificial Intelligence, Southwest Hospital, Third Military Medical University (Army Medical University), Chongqing, 400038 China; 3 Deepwise AI Lab, Beijing Deepwise & League of PHD Technology Co., Ltd, Beijing, 100080 China; 4 Department of Thoracic Surgery, Southwest Hospital, Third Military Medical University (Army Medical University), Chongqing, 400038 China

**Keywords:** Multidisciplinary team consultations, Lung cancer, Artificial intelligence, Medical informatics, Clinical research data platform

## Abstract

**Purpose:**

Multidisciplinary team (MDT) consultations are crucial for managing pulmonary nodules, yet face challenges in efficiency, evidence-based decision support, and data utilization within the MDT process. We present an integrated artificial intelligence (AI)-MDT platform that serves as an assistive tool for lung cancer MDT workflows by incorporating AI across various processes.The aim of this study is to evaluate the clinical utility and preliminary efficacy of the AI-MDT platform.

**Methods:**

The platform comprises three core modules: process automation, intelligent decision support, and diagnostic assistance. It integrates a real-time, evidence-based knowledge base powered by large language models and deep learning, with computer vision for automatic lesion detection and feature analysis. A web-based interface allows users to interact seamlessly with the AI-MDT platform.

**Results:**

Since its implementation in November 2023 at a tertiary Grade A hospital in China, the platform has been involved in 879 consultations, including 811 patients. AI-generated diagnostic recommendations were utilized 852 times, and decision-making support was used in 744 cases. The platform significantly increased consultation volume, reduced expert time, and enhanced data utilization compared to traditional MDT.

**Conclusions:**

It offers clinicians tools to improve diagnostic quality and work efficiency, highlighting its significant clinical application value. These findings suggest that the proposed platform contributes to the emerging research on advances precision lung cancer management by integrating a continually updated evidence base and intelligent imaging methodologies, having potential implications for MDT processes across various medical specialties.

## Introduction

In China, the prevalence of lung nodules increased in lung cancer screening (Long et al. 2023; Yang et al. 2020). Given the increasing detection rate of pulmonary nodules and their potential for malignant progression, early intervention and effective treatment strategies to improve patient survival rates merit attention in both diagnosis and treatment. It is essential to achieve early diagnosis and minimize the risk of overtreatment through a multidisciplinary effort that involves respiratory, thoracic surgery, radiology, and oncology departments. Making optimal diagnostic and therapeutic decisions depends on input from a broad range of clinical specialists. The multi-disciplinary team (MDT) plays a vital role in pulmonary management by bringing together expertise from various clinical teams to enhance pulmonary nodule diagnosis and treatment (Detterbeck et al. 2013; Kowalczyk and Jassem 2020). However, MDTs face several challenges: they struggle with organizing consultation sessions efficiently, have limited access to expert resources, deal with varying expert opinions, and find it difficult to monitor patients throughout their complete treatment cycle.

Researchers have proposed creative solutions to these MDT issues, exploring pathways to improve operational efficiency through both remote approaches and streamlined processes. Specialized remote MDT platforms have been developed for specific diseases (Ewers et al. 2024; Zhang et al. 2023), while others have developed MDT electronic information and communication technology (ICT) tools (Magee et al. 2024). For pulmonary diseases specifically, Vancheri et al. designed a network platform managing idiopathic pulmonary fibrosis through MDT - this platform lets secondary centers send anonymized medical records to tertiary centers for second opinions, making diagnosis both faster and more accurate (Vancheri et al. 2021). To handle cognitive differences among experts within MDT processes and reduce workload, other research suggests developing clinical decision support systems based on clinical guideline rules (Pluyter et al. 2020). By offering evidence-based recommendations for individual patients, these systems relieve team time pressure, organize patient case discussions more effectively, and minimize errors and omissions. These systems now operate in skin cancer (Ali et al. 2024), breast cancer (Patkar et al. 2012), and lung cancer MDT (Kim et al. 2020; Sesen et al. 2014). Existing MDT platforms have attempted and effectively addressed single issues such as inefficiencies in processes, discrepancies in expert opinions, and procedural complexity. However, none of these platforms have been able to integrate multiple advanced technologies while simultaneously meeting various critical clinical needs. This limitation constrains the potential to improve the diagnosis and treatment of lung cancer in primary hospitals and to enhance the survival quality of patients. Moreover, ensuring data security in MDT platforms is critical, especially when incorporating multimodal systems to safeguard patient information (Gawande et al. 2016).

The widespread application of AI technologies in lung cancer management offers potential solutions for MDT to address long-standing issues in the field. Through various cross-methodologies—machine learning, deep learning, computer vision and natural language processing (NLP)—AI advances have enhanced both accuracy and efficiency in diagnosis and treatment. When analyzing imaging, pathology and gene data, studies indicate AI’s power to extract information and utilize it for lung disease diagnosis and prediction. Lightweight convolutional neural networks (CNNs) have been used in thermographic imaging to improve predictive accuracy for oncology, demonstrating their potential for real-time analysis (Thukral et al. 2025). Similarly, robust feature extraction from omnidirectional medical images enhances AI’s diagnostic reliability (Aggarwal and Chauhan 2025). Deep learning has been widely applied in biomedicine, particularly in predicting protein modification sites and their relationships with disease development, significantly improving diagnostic accuracy. Models like PSSM-Sumo for sumoylation site prediction, XGBoost-enhanced ensemble models for feature classification, and Deep-m5U for RNA modification prediction have shown effectiveness in biomedical applications (Khan et al. 2024, 2025; Noor et al. 2024). Not only can AI technology integrate and utilize single model data (Torrente et al. 2018), but it also successfully merges imaging, pathological, and genetic data to diagnose, screen, and predict lung diseases (Echle et al. 2021; Shao et al. 2023; Zhang et al. 2024). Earlier work on light propagation in biological tissues using Monte Carlo and FEM methods also contributed to advancements in non-invasive cancer detection (Kumer 2008). Moreover, evidence suggests the increasing tendency of utilizing AI technology in the development of clinical decision support systems (CDSS), in this case the MDT of lung cancer, which incorporate real-time and personalized features, thus increasing efficiency of workflows (Pluyter et al. 2020). Earlier works have documented that the most commonly utilized methods in CDSS include logistic regression, support vector machines (SVM), fuzzy logic classifiers, and artificial neural networks (ANN) (Fernandes et al. 2020). Research indicates an increase in the emergence of large language models (LLMs) due to the rapid progression of NLP, driven by the breakthroughs in deep learning (Brown et al. 2020). LLMs, trained on immense amounts of textual data, are significant text generators and acquire complex semantic structures in different semantics. This demonstrates significant potential for improving CDSS. This observation has already been demonstrated by other studies, which exhibit that LLMs have already been utilized in CDSS (Liu et al. 2023; Sandmann et al. 2024). They are highly relevant, especially for working with complex medical texts (Dagdelen et al. 2024), communicating critically important information that can promote the clinical decision-making process. Of note is the ability of LLMs to manage massive text data quickly, facilitating the reduction of the time taken to conduct literature searches and increasing the accuracy of information extraction.

When applied to lung cancer management, these technologies boost overall efficiency and facilitate MDTs in better coordinating and integrating expert opinions across various fields, leading to more accurate and effective treatment decisions (Ansoborlo et al. 2023). By taking this approach, AI delivers strong support for personalized lung cancer treatment, solving issues observed in traditional management methods including management and integration of complex data, multiple data sources coordination, and uncertainty in clinical decision-making (Ladbury et al. 2023). However, the complexity of algorithms make clinicians struggle to use them independently for analysis. One previous study addressed this challenge by creating advanced algorithm-based medical imaging analysis tools, giving clinicians an easy-to-use AI toolkit ( Wang et al. 2024). In addition to imaging information, our platform automatically applies AI algorithms to multimodal data and generates diagnostic and predictive outcomes, assisting clinicians make more accurate decisions.

Thus, our team has developed an AI-MDT artificial intelligence-assisted decision-making platform specifically for lung cancer. This platform pulls heterogeneous data from both inside and outside the hospital, builds an evidence-based medical library, and offers diagnostic and treatment recommendations based on physisans verification—all while enabling intelligent diagnosis and staging to strengthen MDT decision support assistance. A comprehensive patient management platform was developed to address the patient lifecycle’s need for data tracking and management for smooth integration and information sharing. The research’s objectives were to improve the efficiency and quality of the MDT’s decision, promote optimization of lung nodule management strategies, and enhance patient treatment through AI-MDT platform application.

## Requirement analysis

### The need for automation in the MDT consultation process

Specifically, the traditional MDT consultation process is burdened with the administrative load to the extent of being inefficient. With the advancement of medical informatization, hospitals have developed various information systems in which different information systems were deployed, such as HIS (Hospital Information System), LIS (Laboratory Information System), and RIS (Radiology Information System). Nevertheless, data integration continues to remain a problem. Therefore, physicians have to spend significant time alternating between sources in gathering and processing data prior to an MDT consultation, which reduces their productivity. The MDT consultations of the past suffered from limited onsite data mining resources, meaning that even the most basic of decision-making and evidence acquisition was carried out during the consultation, which only prolonged and complicated the entire procedure. Even after consultations end, medical staff face the task of manually creating and submitting MDT reports, often producing inconsistent, non-standardized content that proves less useful for future reference. These inefficiencies clearly exhibit why we urgently need to automate and structure the entire MDT process.

### The need for establishing an evidence-based knowledge base

As lung cancer diagnosis and treatment evolves rapidly, medical experts must constantly keep up with new clinical and translational research findings. However, physicians struggle to stay current with the latest academic developments due to evidence-based medical information consists of a vast, diverse range. This challenge becomes especially acute during advanced treatment stages that lack established guidelines such as third-line therapies for advanced lung cancer. When second-line treatments fail at this stage, patients face tough choices as no guidelines exist to offer evidence-based support. Therefore, it is critically important that a decision support platform be developed by integrating big data and artificial intelligence to address this issue; and accordingly, a real-time, evidence-based knowledge base can offer doctors with up-to-date, evidence-based support through cutting-edge diagnostic and treatment recommendations.

### The need for effective utilization of data mining

Medical data often involves broad scope of information regarding patients’ diseases, and this especially holds true for radiology data. However, the efficient and accurate information extraction represents a significant expenditure of time for physicians, and often leads to inefficiency MDT consultations, hence rendering AI technology vital in the process of MDT consultations in particular when information exceeds human processing capabilities. Therefore, it is necessary to integrate AI in the process of intelligent lung nodule analysis on lung cancer MDT platforms to enable to rely on AI integrated platform for data extraction than having to manually write complex code or perform calculations before consultations. It can be argued that, this new approach can significantly reduce the time expenditure and enhance work efficiency for image review.

While online platforms have been developed in certain hospitals to improve the efficiency of MDT consultations, they were mostly targeted for specific tasks, such as streamlining the Multi-Disciplinary Meeting (MDM) process, enabling remote MDT scheduling, automating evidence-based decision-making for skin cancer, and supporting asynchronous discussions of medical conditions through cloud platforms. To propel MDT development forward, it is necessary to establish an integrated platform that combines big data with AI technologies. Such a platform would automate MDT processes utilizing big data, deliver evidence-based recommendations, and analyze multimodal diagnostic and treatment data in detail. Through this approach, we aim to build a comprehensive, cutting-edge, and user-friendly MDT platform that both simplifies consultations and enhances MDT management.

## Materials and methods

### Overall platform architecture

**Fig. 1:**
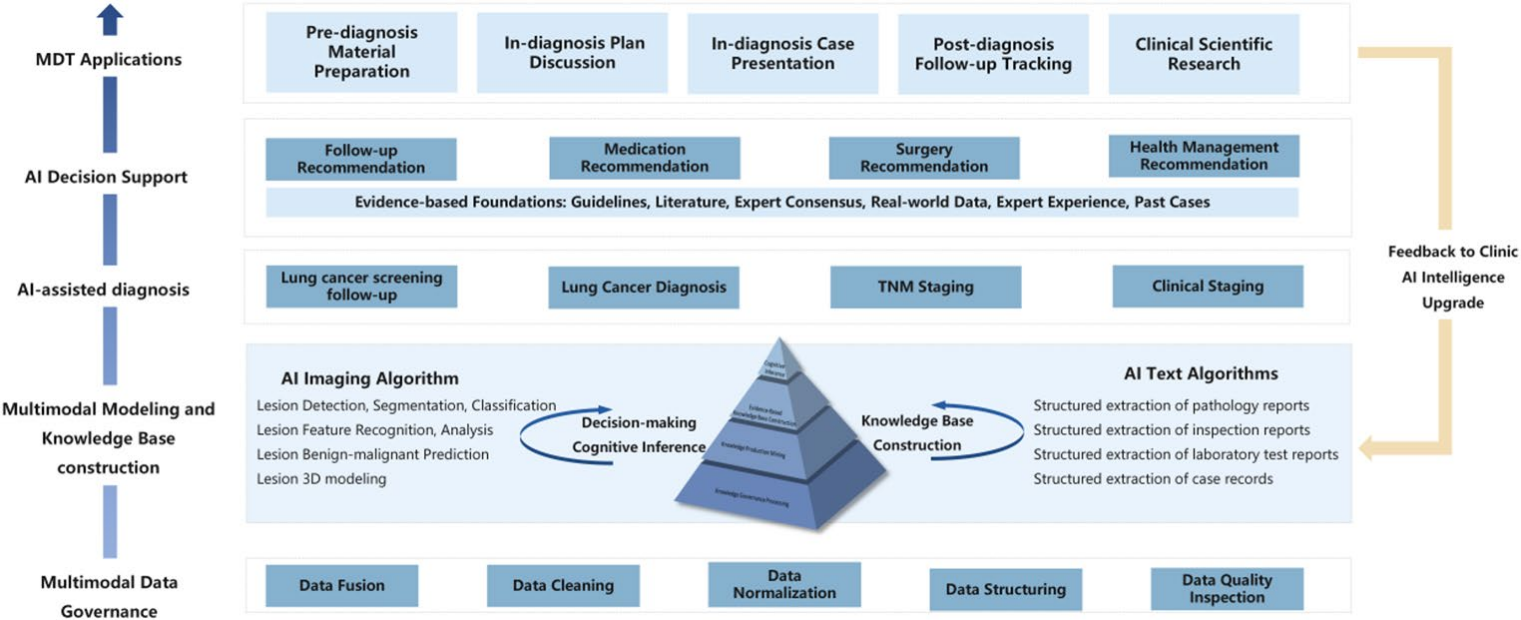
Framework of AI-MDT platform

The platform follows a modular, hierarchical design built on a rack server running CentOS, with Kubernetes handling management and Docker containers operating on a hyper-converged virtual platform—all working collectively to pool computing and storage resources. From bottom to top, five layers make up the structure: multimodal data governance, multimodality modeling and knowledge construction, AI-assisted diagnostics, AI decision support, and the MDT application layer. And the framework of AI-MDT platform displays in Fig. 1.

Starting at the foundational level, the data governance layer handles complex, scalable hospital data through Hadoop and Ceph-based big data distributed storage. This layer pulls patient data from various information systems, utilizing database synchronization and ETL (Extract, Transform, Load) to merge and clean data. One level up, the multimodal modeling and knowledge construction layer uses NLP to extract clinical information features for predictions, offering algorithm services through an application programming interface (API). Here, a Transformer deep learning framework supports an evidence-based knowledge base storing clinical information. Built on this foundation, the AI-assisted diagnostic layer and AI decision support layer work in tandem. The diagnostic layer employs deep learning networks to pull semantic features from medical images and correlate them with clinical indicators for comprehensive diagnosis. Meanwhile, the AI decision support layer delivers intelligent, personalized diagnostic and treatment recommendations based on both the evidence-based knowledge base and AI-extracted information. At the top, the MDT application layer integrates all layers, constantly improving through feedback mechanisms. This progressive structure creates a complete cycle - from data processing straight through to clinical application.

### AI-based MDT automation process

The AI-MDT platform seeks to automate the entire MDT consultation process to help physicians work more efficiently while ensuring data accuracy. We built the platform utilizing Hadoop analytical architecture, B/S browser architecture, and big data search engines, which offer distributed storage for both semi-structured and unstructured clinical and medical imaging data. This setup also supports distributed computing for NLP and computer vision algorithms.

**Fig. 2:**
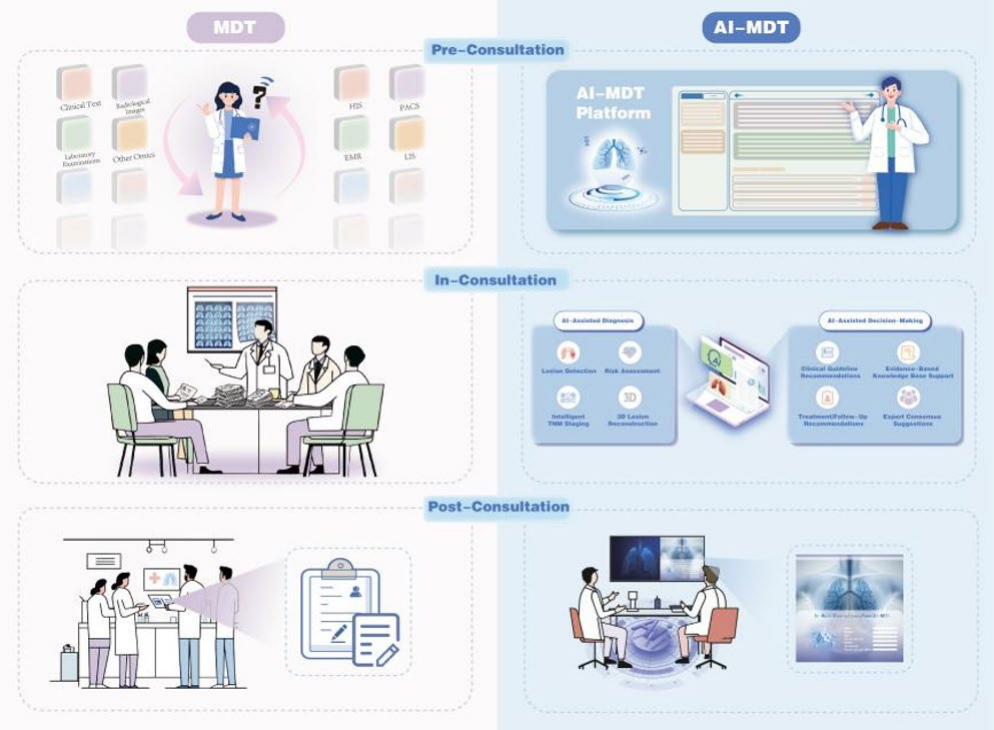
Comparison of AI-enhanced and traditional MDT platforms within MDT process: workflow optimization from pre-consultation preparation to post-consultation management

We have innovatively introduced the AI-MDT platform, which integrates advanced AI technologies, including LLMs, deep learning, and computer vision. This integration enables workflow automation, provides intelligent diagnostic and decision-making support, and enhances data analysis capabilities. The platform’s AI-assisted process spans three stages from pre-consultation to post-consultation, progressively enhancing the accuracy and explainability of system outputs. First, in the image analysis stage, AI integrates serial imaging data, assisting radiologists in lesion identification and suspicious case screening, only incorporating patient information confirmed by human verification into subsequent processes. Second, during the clinical information integration stage, AI automatically combines multidimensional information including medical history, family history, pathology, and genetic testing results based on physician-confirmed imaging diagnoses to form structured medical records for physicians to verify. Finally, in the decision support stage, AI retrieves and matches authoritative domestic and international guidelines based on physician-confirmed final diagnoses, generating recommendations with clear supporting evidence. Final diagnostic and treatment decisions are made collaboratively after thorough discussion by at least five senior MDT experts and reviewed by the primary specialist. Through the closed-loop mechanism of “AI assistance—physician confirmation—knowledge base tracing—MDT revalidation,” the platform ensures that diagnostic and decision-making processes are clinically reliable, logically explainable, and medically traceable, enabling safe and trustworthy application of AI technology in real clinical scenarios. Compared to traditional MDT methods, our platform streamlines the entire lung cancer MDT workflow, from pre-consultation to post-consultation (Fig. 2).

#### Multi-source heterogeneous data governance

The system automatically retrieved patients’ inpatient medical records and selected imaging data, providing the data foundation for physician information preparation. The platform governed multi-source heterogeneous data. Multi-source heterogeneous data processing technology, deployed on big data computing cluster servers, can rapidly identify the structure of data backed up from hospital front-end machines and quickly establish associations and analyses between different data tables, achieving data integration. Data integration is conducted across patient dimensions, visit dimensions, and medical order dimensions, and through computational models and various data element standards, the data undergoes cleaning and processing. Notably, medical imaging data is collected and processed according to the standard DICOM 3.0 protocol. Meanwhile, text-based data such as clinical information and laboratory results requires processing based on its specific semantic content. This processing typically includes data cleaning, normalization, standardization, structuring, and completion, among other routine data management procedures.

#### Data security and privacy

When developing modern medical information systems, protecting data security and privacy stands as a crucial priority. Our platform addresses this issue through several approaches. We apply encryption algorithms (e.g., AES and SCB2) during data storage and transmission, managing encryption keys through a centralized system. Based on user roles and operational needs, we maintain strict data access control with full logging and auditing capabilities. For network security, we have implemented firewalls and bastion hosts in the hospital’s intranet while utilizing SSL technology for additional protection. The platform is deployed on local private servers within the hospital, with complete physical isolation from external networks, ensuring all data processes remain within the internal environment. Additionally, the large language models used by the platform are locally deployed based on open-source frameworks, without relying on external cloud services, thus avoiding the risk of external data leakage during model invocation. Model updates and optimizations are implemented only after in-hospital manual review and offline testing, ensuring safety and controllability.

Following internationally recognized HIPAA regulations and complying with the Personal Information Protection Law and Data Security Law,, our platform employs various anonymization algorithms (including masking, distortion, replacement, randomization, format-preserving encryption, and strong encryption algorithms) to handle sensitive data. We apply both Static Data Masking (SDM) and Dynamic Data Masking (DDM) to meet different scenario requirements.

The platform’s development and clinical use have received approval from the hospital’s ethics committee and information security committee, with multidisciplinary data access following the principle of minimal authorization. Additionally, the platform deployment environment complies with the National Information Security Classification Protection (Level 3) and ISO 27,001 Information Security Management System standards. We conduct regular vulnerability scanning, security audits, and data backups to ensure the system remains stable, secure, and compliant during long-term operation.

### Establishment of an evidence-based knowledge base using LLMs

In CDSS, LLMs have been gaining traction through extensive language processing capabilities, offering new pathways to handle increasingly complex and voluminous medical literature. In this module, we have incorporated a medical LLM module into structured data management to create intelligent diagnostic and decision-making recommendations. This approach minimizes time spent by doctors on searching through extensive literature and guidelines, and enhances both standardization and quality in post-diagnostic clinical decisions.

This study developed an LLM-based MDT-assisted decision recommendation module. It builds an evidence-based knowledge base according to clinical needs and enables automated analysis and matching of evidence materials to support clinical decision-making. First, AI-MDT automatically updates the information related to the latest clinical guidelines, expert consensus, clinical trial results, and literature reviews based on evidence drawn from medically endorsed websites. Such reviews are automatically parsed utilizing an LLM powered by GPT-4 technology. The core technology is a neural network model based on the architecture of transformers, which utilizes self-attention and encodes position to capture dependencies in language structure that are long-range.

Mathematically, the self-attention mechanism is defined as

1 $$\:Attention\left(Q,K,V\right)=softmax\left(\frac{Q{K}^{T}}{\sqrt{{d}_{k}}}\right)V$$

where $\:Q,K,V$ represent the query, key, and value matrices derived from tokenized inputs, and ​$\:{d}_{k}$ denotes the dimensionality of the key vectors for scaling. The scaled attention scores are normalized with a softmax function to convert them into probabilities (attention weights).

This formulation computes pairwise relevance between tokens in a medical narrative, allowing the model to assign higher weights to clinically important terms (e.g., linking “spiculated nodule” with “adenocarcinoma cells” and “bloody sputum”). Such attention weighting enables the prioritization of critical patient information during automated recommendation generation, improving both accuracy and context sensitivity.

The platform has been locally deployed and further developed based on open-source large language model frameworks, primarily utilizing prompt engineering and retrieval-augmented generation with knowledge graphs to achieve medical report interpretation, multidisciplinary knowledge integration, and meeting summary generation. During the text generation, the model predicts the next word in a sentence coherently formed with an already given context. This LLM was further employed in extracting key information such as experimental design type, trial phase, driving genes, Performance Status Score (PS), treatment stage, and experimental group treatment protocols. All the information retrieved was subjected to rigorous review by professional physicians to ensure the validity and reliability of the information. The verified data was then incorporated into the MDT-assisted decision-making recommendation module. Besides, the system advances and updates the knowledge base derived from updates from sources such as guideline consensus to enable all physicians to utilize the latest standardized treatment decision-making protocols. The final iteration of the knowledge base for the treatment decision-making regarding lung cancer is deployed in a hospital and implemented with a lightweight search engine algorithm to guarantee the efficiency of response times.

When applying this module in clinical settings, we match patient-specific details with clinical trial articles to make recommendations as below: for each piece of literature, we match the patient’s data (e.g., TNM staging, driving genes, PD-L1 expression levels, PS, and treatment stage) against matching information in the respective article. Our set of scoring criteria gives one point for each perfectly matched field, then adds these points collectively to calculate how well the literature matches the patient’s case. We then rank and recommend articles based on these total scores, helping clinicians quickly find accurate information that fits each patient’s unique situation.

### Deep learning-based imaging analysis module

**Fig. 3:**
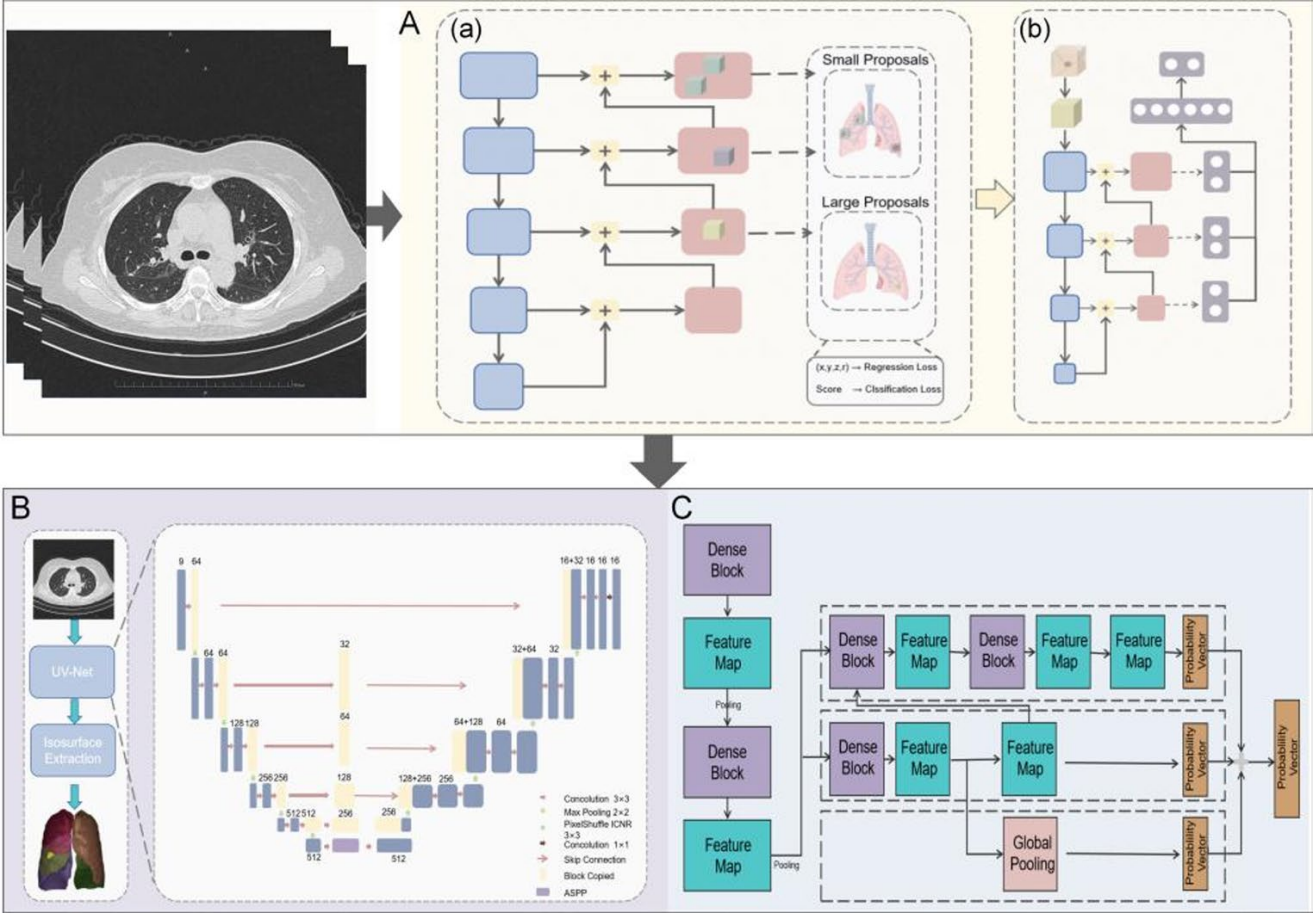
Workflow of network architectures in deep learning-based imaging analysis module. **A** Lung lesion identification using a pipeline model for lung nodule detection: first, candidate regions are extracted (a), followed by false positive reduction (b). **B** 3D reconstruction of the segmented image using UV-Net and Isosurface extraction. **C** Classification network with a 3D neural network algorithm employing multiscale feature fusion for image-to-feature extraction

Recent progress in machine learning and deep learning has transformed medical image processing, and allowed for the application of AI technologies in several aspects including detecting pulmonary nodules (Sun et al. 2018), reconstructing three-dimensional lung images (Wang et al. 2021), and diagnosing lung cancer through medical imaging. The network architectures of each module are illustrated in the Fig. 3. The AI-MDT platform employs various neural network algorithms. Neural networks are highly non-linear systems whose complexity varies according to the number of neurons and layers.

To provide a unified mathematical foundation for the deep learning methods utilized in our platform, we describe the training of neural networks for medical image analysis as an optimization problem:

2 $$\:{\theta\:}^{\ast\:}=\text{arg}\underset{\theta\:}{min}\:\frac{1}{N}\sum\:_{i=1}^{N}\:\mathcal{L}\left(f\left({X}_{i};\theta\:\right),{Y}_{i}\right)+\lambda\:\mathcal{R}\left(\theta\:\right)$$

where $\:{X}_{i}$ denotes the input data (e.g., CT slices, 3D volumes, or extracted features); $\:\theta\:$ represents all learnable parameters of the neural network model $\:f(\cdot\:)$; $\:{Y}_{i}$ is the ground truth label for $\:{X}_{i}$, which varies depending on the specific task (classification, detection, segmentation); $\:\mathcal{L}\left(f\right({X}_{i};\theta\:),{Y}_{i})$ is the loss function quantifying the difference between model predictions and ground truth; $\:\mathcal{R}\left(\theta\:\right)$ is a regularization term (such as L2 or L1 regularization) to prevent overfitting; $\:\lambda\:$ is a hyperparameter that balances the loss and regularization terms.

This mathematical framework applies to all major tasks in our platform: For classification, $\:{Y}_{i}$ is a class label (e.g., benign or malignant). For object detection, $\:{Y}_{i}$ consists of bounding boxes and class labels. For segmentation, $\:{Y}_{i}$ is a pixel-wise or voxel-wise mask. During training, optimization algorithms such as SGD or Adam are used to iteratively update $\:\theta\:$ and minimize the overall loss. This unified approach underpins the specific network architectures and modules described in the following sections, ensuring both accuracy and generalizability in medical image analysis.

#### Lung nodule identification

For detecting lung nodules, greater challenges are present than traditional natural image detection as nodules appear small and exhibit subtle texture differences. The challenge grows even more complex since CT machines vary in models, dosage levels, and reconstruction algorithms - creating significant deviations in CT data distribution, which necessitate enhanced generalizability of our model.

Our pipeline model for lung nodule detection operates in sequential phases. The initial phase identifies potential nodule regions of interest, followed by false positive elimination (Fig. 3A). For candidate region extraction, the model has implemented a U-Net segmentation framework based on a feature pyramid, enhancing the network’s capability to identify and differentiate small nodules across multiple resolutions. The network’s sensitivity to smaller nodules improves through minimized anchor size and optimized anchor matching methods. An optimized 3D ResNet50 acts as the model’s backbone, incorporating dilated convolutions instead of pooling layers to enhance minute nodule detection. The output phase employs a dual-output model structure: first predicting nodule probability at each pixel, then calculating a four-dimensional offset vector relative to nodule centers. This architecture integrates a combined loss function, utilizing Focal Loss for classification and Huber Loss for regression, calibrated to ground truth labels. This configuration enhances both detection accuracy and network generalizability for lung nodules. The final phase implements a U-shaped classification network to eliminate false positives, ensuring optimal nodule detection accuracy.

#### Three-dimensional reconstruction

Based on the segment algorithm, the module conducts three-dimensional (3D) reconstruction. The foundation of the module incorporates a deep learning 3D lung segmentation algorithm, utilizing UV-Net as its primary architectural backbone (Fig. 3B). In its U-shaped network design, 2D encoder modules integrate with 3D decoder modules, allowing adjustments based on specific data properties. The implementation of an Atrous Spatial Pyramid Pooling module expands the model’s receptive field while integrating global contextual information, enabling multi-scale feature map fusion that preserves both spatial and semantic details. During upsampling, the model employs a sub-pixel (PixelShuffle) methodology to restore feature map spatial resolution, thus maintaining optimized details and achieving comprehensive topological structure continuity in pulmonary vessels and airways. The segmentation process comprises multiple components: the right upper, middle, and lower lobes, plus the left upper and lower lobes of the lungs, with accurate boundary between segments. The tracheal model segmentation preserves the dendritic structure and connectivity of airways, while the arteriovenous model differentiates and segments the complete network of pulmonary blood vessels into arterial and venous components. These segmented datasets then facilitate 3D reconstruction processes.

#### Prediction of malignancy and feature recognition of lung lesions

The network framework, following lesion identification from the preceding module, employs a 3D neural network for CT-based benign and malignant lung nodule differentiation, specifically structured to optimize adaptation to lung nodule anatomical structure (Fig. 3C). The base network’s design incorporates a multi-scale feature fusion approach, orchestrating three yet complementary networks: the local branch network extracting nodule-specific local features, the middle-layer branch network capturing surrounding contextual features, and the global branch network extracting broader structural elements. To achieve enhanced model effectiveness, this study implemented a discriminant filter network specifically onto the base networks of both local and middle-layer branches, whereby this additional network performs detailed image block feature mining while supplementing discriminative information elements. The systematic process is through three sequential phases: (1) The backbone network executes comprehensive feature extraction from lung nodule image data, generating both general prediction probabilities for benign/malignant classification and overall nodule density type probabilities. (2) A dedicated local feature extractor processes the image data to extract local features, calculating specific local prediction probabilities for both nodule type and density parameters. (3) Through the established connections between multi-scale feature maps—generated through local feature pyramid analysis—the system derives final benign/malignant prediction probabilities by synthesizing all previous probability calculations.

#### cTNM staging

Medical images are processed and image features are extracted with the algorithms from the previous modules, while NLP simultaneously extracts information from the patients’ electronic medical records, including lung nodule size, regional lymph node metastasis status, distant metastatic lesion presence, and other characteristics. The platform integrates deep learning models with rule-based methods, whereby ensemble learning strategies merge various heterogeneous intelligent analysis models into a unified decision-making model for lung cancer TNM staging. For T staging determination, CT images offer data through which imaging algorithms accurately calculate lesion size and invasiveness, following the AJCC 8th edition guidelines to classify T1 through T4. N staging determination proceeds through comparison of primary tumor and lymph node relationships (whether ipsilateral or contralateral), combined with electronic medical record data to determine N1 or N2. M staging relies on NLP algorithms extracting relevant information from radiology reports to identify metastasis presence.

### Operational process of the AI-MDT platform

**Fig. 4:**
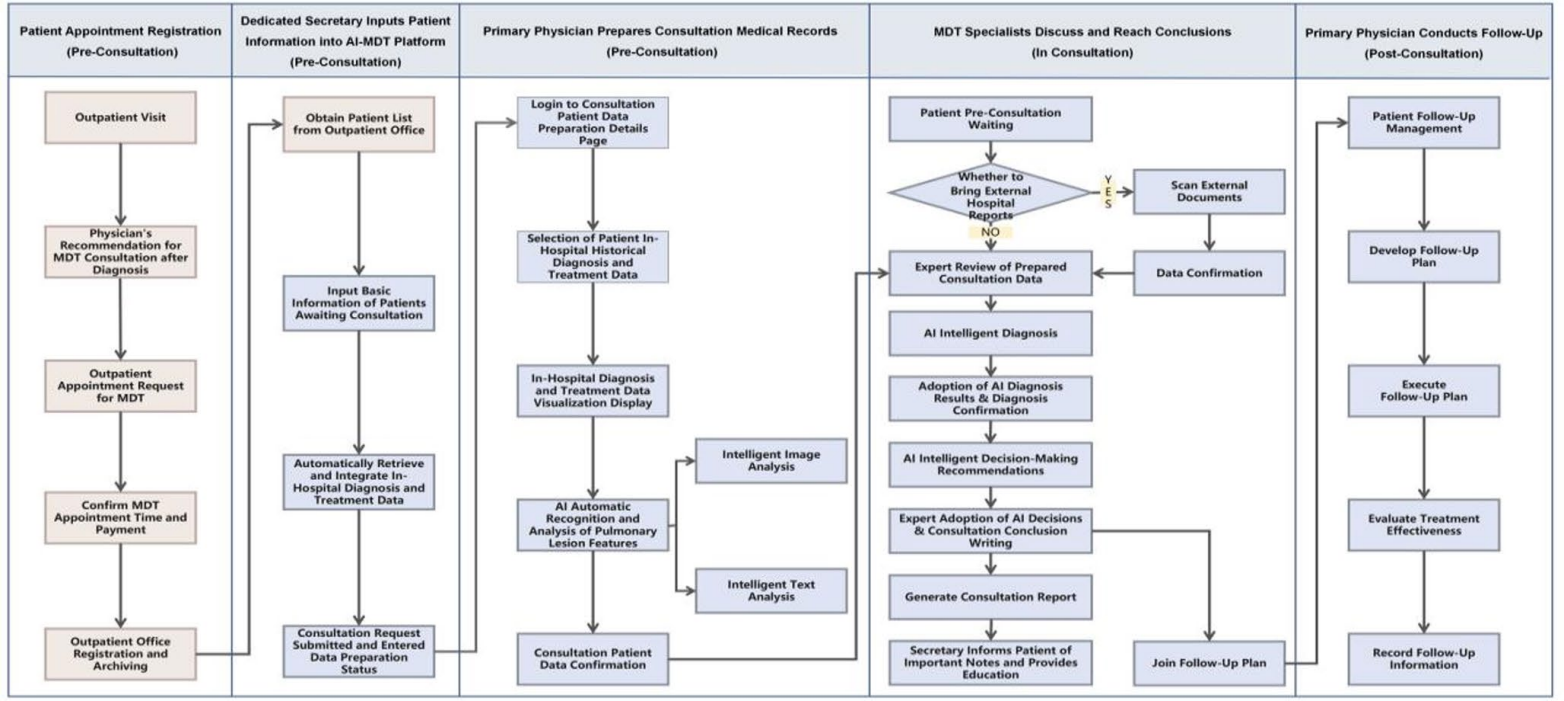
Workflow scheme of AI-MDT platform

The study designed the process of AI-MDT consultation (Fig. 4). After an appointment is scheduled, the platform automatically collects and integrates both internal hospital medical data and patient-provided information. Through the platform, physicians can easily access all necessary MDT consultation data and pick the specific information they need for pre-consultation preparation. In the decision-making phase, our platform produces AI-enhanced clinical recommendations, each recommendation linked to corresponding evidence-based guidelines, ensuring complete transparency and traceability. Following physician validation of the final diagnosis, the system searches and aligns with leading clinical guidelines from both domestic and international guidelines to generate recommendations with clear traceable evidence. The final clinical decisions emerge from interdisciplinary collaboration among at least five experienced MDT specialists, who engage in comprehensive case discussion before reaching consensus. These decisions are subsequently reviewed and formally endorsed by the lead specialist overseeing the case. Based on this collaborative approach, they can generate detailed, structured consultation reports with a single click - rendering it simpler to plan follow-ups, treatments, and health guidance.

To manage potential discrepancies between AI-generated suggestions and final MDT decisions, our platform implements a comprehensive traceability framework. Each AI recommendation is associated with its source evidence. Cases involving inconsistencies are documented for retrospective analysis to determine the root cause whether arising from procedural errors, data limitations, or ambiguities in guidelines. This systematic review process supports continuous refinement and optimization of the AI-MDT system.

## Results

### Application usage

This study, through collaborative support from the hospital’s big data center, multiple departments, and medical informatization construction companies, has established and implemented the aforementioned AI-MDT artificial intelligence-assisted decision-making platform for lung cancer in a Grade III Class A hospital in China, according to the described design and implementation methods.

**Fig. 5:**
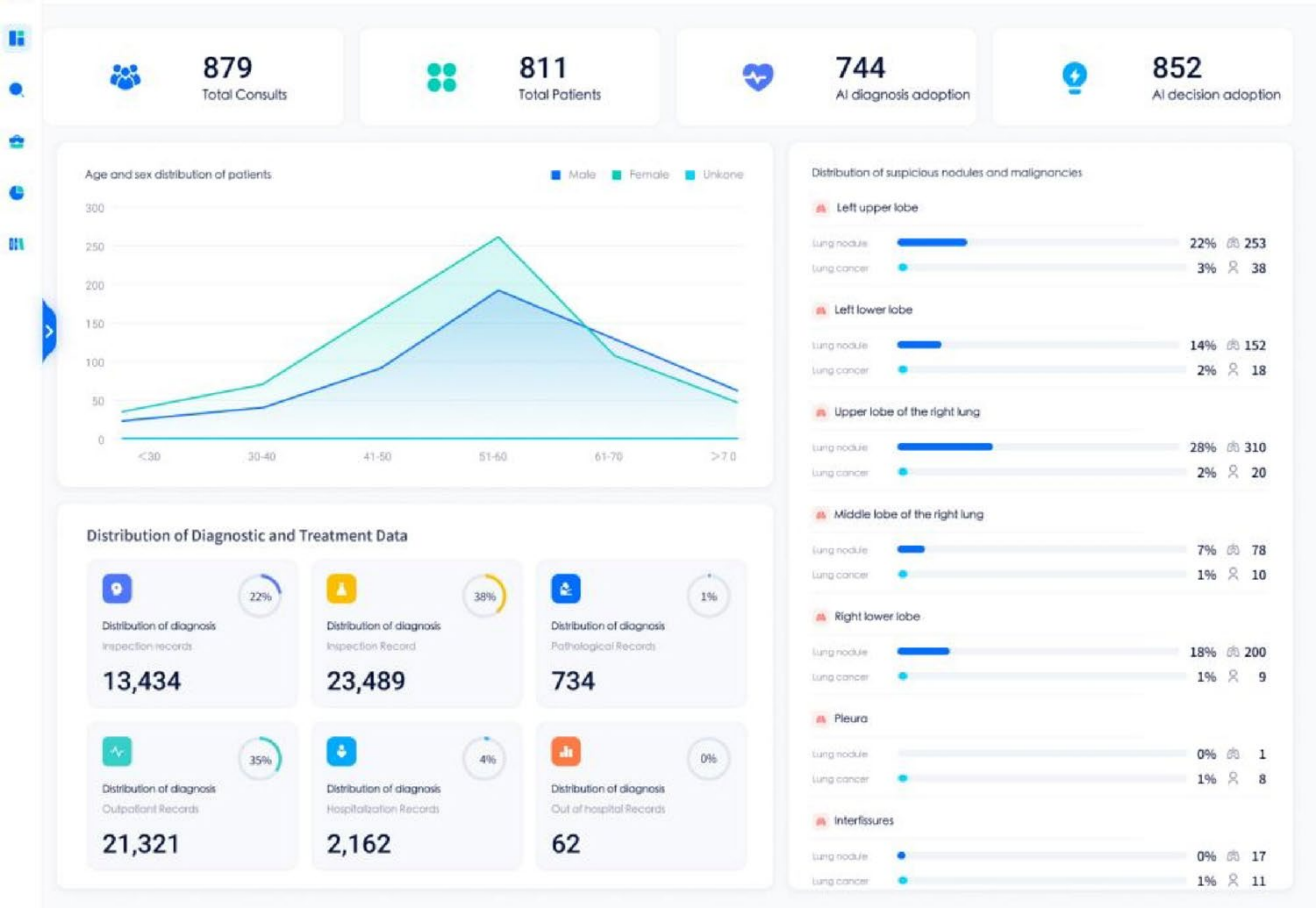
The user interface of the proposed AI-MDT platform

Since the AI-MDT platform’s launch in lung cancer MDT clinic on November 14, 2023, it has facilitated a total of 879 consultations with 811 patients. These cases saw 852 adoptions of AI diagnostic suggestions and 744 adoptions of AI decision supports. The Fig. 5 demonstrates the AI-MDT platform interface for patients with pulmonary nodules or cancer.

### The data governance module shortens the Preparation time for multidisciplinary consultation materials

**Fig. 6:**
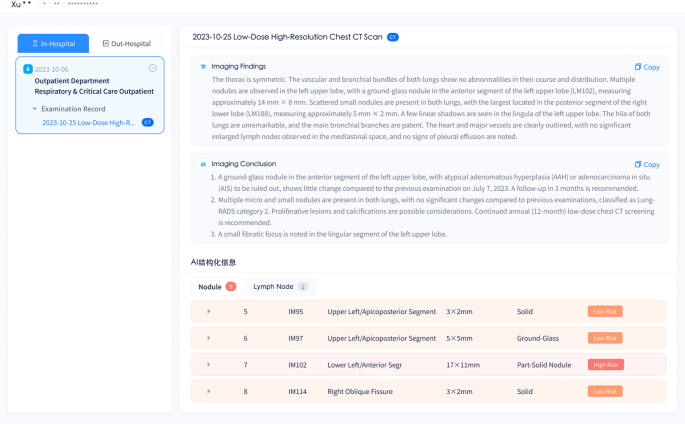
Interface for pre-consultation patient data preparation

The platform automatically gathers key clinical information, highlights risk factors, and provides a patient overview. Physicians can select relevant data to support multidisciplinary consultations. Upon MDT consultation application, the assistant physician utilizes the platform for relevant patient data selection, whereby consultation information preparation completes through simple option selection checkmarks (Fig. 6).

### The evidence-based knowledge base and intelligent imaging analysis module provides a one-stop solution for diagnostic assistance and decision support

#### Decision recommendations

**Fig. 7:**
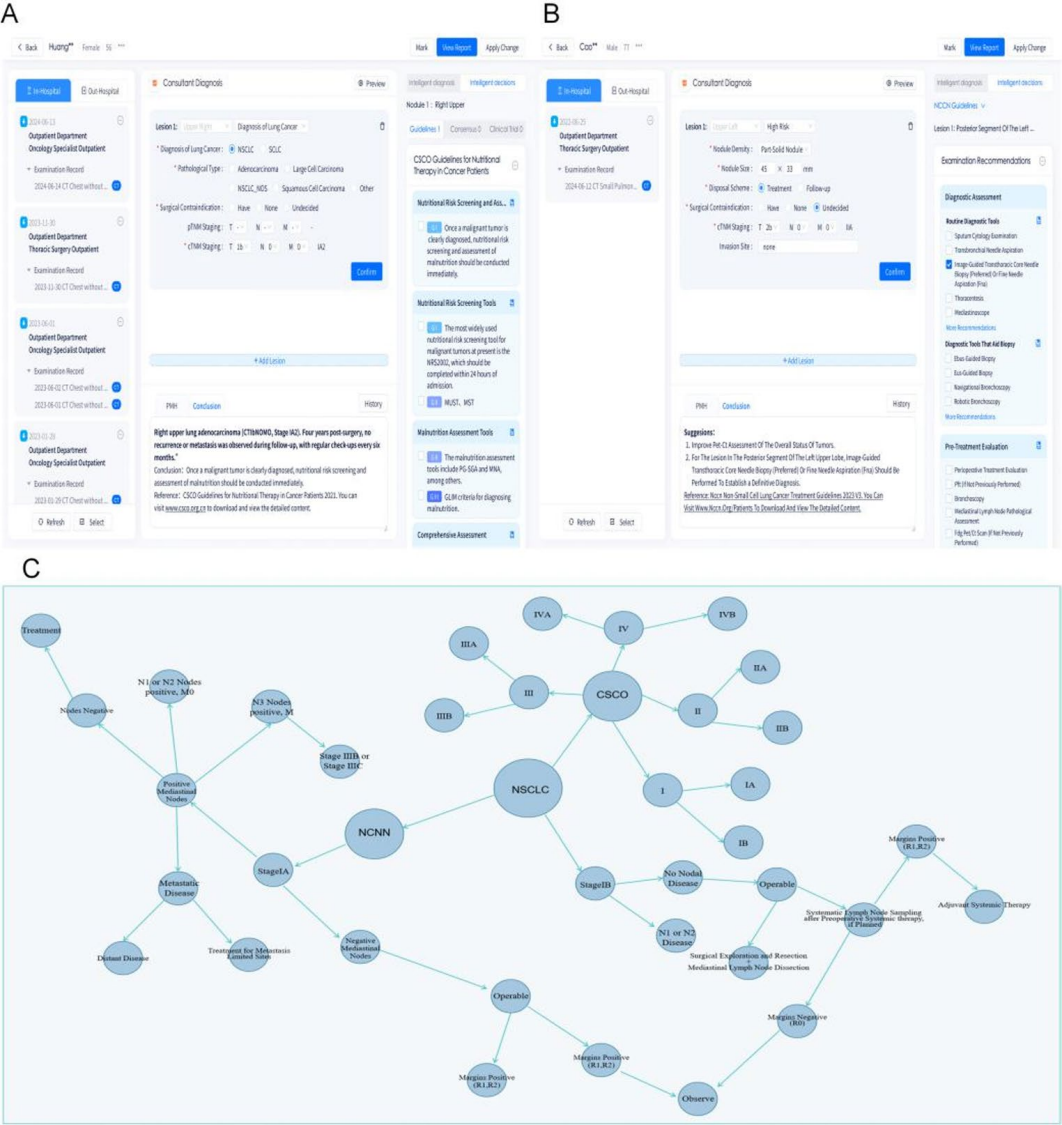
AI-MDT platform for personalized lung cancer diagnostic and treatment pathways and knowledge graph integration. **A**, **B** Illustrations of two clinical scenarios managed by the AI-MDT platform: **A** a first-time patient without a prior diagnosis of malignant lung tumors and **B** a follow-up case post-diagnosis. **C** Visualization of the lung cancer diagnostic knowledge graph embedded within the AI-MDT platform

Based on physician-confirmed imaging diagnoses and structured medical record information, the AI-MDT platform retrieves and matches evidence-based knowledge base information to generate personalized diagnostic and treatment recommendations with traceable evidence. Following thorough discussion among at least 5 senior MDT specialists and final review by the primary specialist, the team formulates the diagnosis, treatment decisions, and follow-up plan.  Fig. 7A and B illustrate two patient scenarios where one was initially undiagnosed with malignant lung tumors, and another was re-examined after being diagnosed with malignant lung tumors. Through evidence-based knowledge base integration, the AI-MDT platform proposes targeted plans: diagnostic assessment and treatment suggestions for first-examination undiagnosed patients, while prioritizing health management plans for post-diagnosis reexamination patients. The decision-making module incorporates multiple functionalities: evidence-based reasoning visualization, knowledge base diagnosis and treatment plan location, automatic consultation report population, alongside reference details and patient-accessible download links. Fig. 7C demonstrates the platform’s established lung cancer diagnosis knowledge graph, wherein decision-making criteria align with guide consensus across different nodes.

#### Intelligent diagnosis

**Fig. 8:**
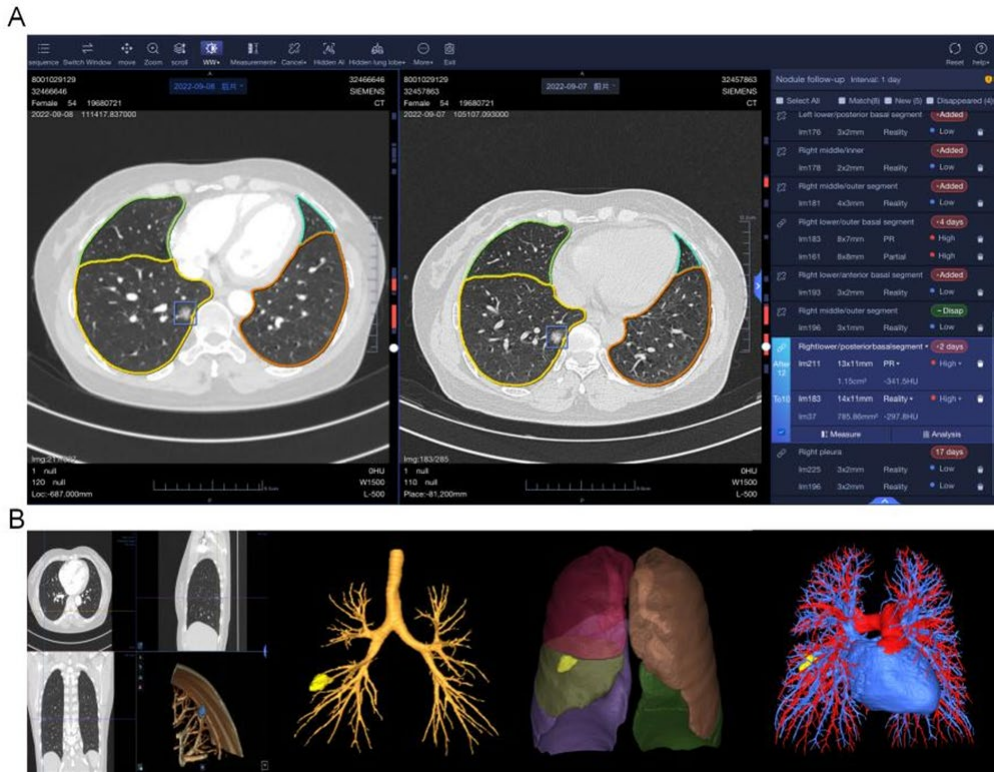
Automated pulmonary image analysis and intelligent risk assessment in the AI-MDT platform. **A** The platform enables automated pulmonary segmentation and highlights high-risk nodules, achieving intelligent risk stratification of pulmonary nodules. **B** The 3D browsing module offers coronal and sagittal reconstructions, rotation, and synchronized display, as well as 3D reconstructions of pulmonary nodules, bronchi, pulmonary lobes and segments, and blood vessels, facilitating multi-angle observation of lesions by clinicians

The AI-MDT platform, as demonstrated in Fig. 8A, executes automatic lung segmentation, identifies potential lung nodules, and implements highlight functionality for physician alerts regarding high-risk nodules. Through radiology algorithm implementation, the platform executes nodule categorization into high, medium, or low-risk classifications, presenting these in the intelligent diagnostic lesion list alongside comprehensive details including location, density, and size parameters, thereby enabling efficient risk evaluation processes for medical practitioners. The platform further incorporates automatic calculation and display capabilities for lung cancer clinical staging through the cTNM staging module. In the 3D browsing module, interactive functionalities consist of multi-planar reconstruction (MPR), maximum intensity projection (MaxIP), minimum intensity projection (MinIP), point measurement, angle measurement, and ROI measurement capabilities. Physicians have access to coronal and sagittal reconstructions, along with 3D lung reconstructions that include rotation and synchronized display options, enabling nodule examination from multiple angles (Fig. 8B). The platform enables physician transfer of AI-diagnosed lesion information into the manual diagnostic correction list, incorporating AI diagnostic suggestions throughout this process. For characteristics beyond platform diagnostic capabilities, physicians retain manual information addition functionality. The platform generates automatic treatment recommendations based upon physician-confirmed final diagnoses. Notably, this module assists radiology specialists in screening, with only patient imaging information that has been manually confirmed being included in subsequent processes.

### Performance statistics

The effectiveness evaluation of the AI-MDT platform comprised a comparative analysis between two groups. The first group was a study group comprising 879 patients who attended lung cancer AI-MDT outpatient consultations following platform implementation (November 2023 to October 2024). The second group was a control group consisting of 459 patients from traditional MDT consultations prior to system launch (November 2022 to October 2023). Statistical analysis analyzed evaluation metrics before and after AI-MDT platform application in the AI lung cancer MDT outpatient setting, specifically focusing on consultation duration and work efficiency parameters. In this analysis framework, consultation duration measures individual patient average visit time allocation, while work efficiency measurements derive from patient volume processing during each outpatient consultation period. Research findings demonstrated that AI-MDT platform implementation produced statistically significant improvements, reflected through reduced consultation duration and enhanced consultation efficiency metrics (Table 1).

**Table 1:** Comparison of consultation duration and work efficiency between traditional and AI-assisted MDT platforms.

	Traditional MDT	AI-MDT	t	*P*
Number	7.29 ± 3.93	19.11 ± 4.12	15.097	< 0.001
Time (h)	0.793 ± 0.866	0.117 ± 0.069	− 6.1649	< 0.001

## Discussion

The decision-making complexity in lung cancer management continues intensifying through exponential growth in diversified medical data and treatment options. High-quality MDT collaboration and coordination remains essential for timely and accurate diagnosis and care delivery. This study introduces an AI-based online platform hosting lung cancer (nodules) MDT consultations, whereby the platform integrates multi-disciplinary medical data and leverages medical AI technology with multiple model algorithms including imaging AI to assist the physician in making the diagnostic decisions.

The user-friendly AI-MDT facilitates streamlined data preparation processes, thereby reducing pre-MDT consultation preparation time requirements. Traditional MDT consultation preparation comprises time-intensive collection of relevant comprehensive clinical information (including essential historical images and clinical data, etc.), necessitating organization into structured, readable formats (Jalil et al. 2013; Janssen et al. 2018; Powell and Baldwin 2014). The significant increase of MDT consultations workload has imposed unprecedented time and efficiency demands upon team members (Kane et al. 2007), prompting wider implementation of digital solutions and management strategies to address these requirements. Research demonstrates that systems integrating all relevant clinical data into single-source have successfully reduced pre-MDT consultation preparation time across multiple cancer types, including breast cancer, gastrointestinal cancer, and head and neck cancer (Hammer et al. 2020). Besides, Merker et al. developed a pre-preparation MDT template for breast cancer that enables real-time patient and information addition capabilities for all clinical staff (Merker et al. 2023). In this study, the AI-MDT platform incorporates automatic extraction functionality for patient in-hospital diagnostic and imaging data while simultaneously integrating external data sources, targeting comprehensive full-process automation. Through optimization of data preparation protocols, structured presentation of key medical information, and implementation of convenient retrieval and selection mechanisms for necessary information, the platform enables medical practitioners to efficiently complete all pre-consultation preparatory requirements.

The AI-MDT platform maintains a vital role in lung cancer management, whereby knowledge graphs and LLMs enable construction of an evidence-based CDSS, offering accurate decision support to physicians. Moreover, algorithms were integrated during the development of the platform to facilitate doctors in making informed judgments, thus enhancing diagnostic accuracy and efficiency. AI has gained some currency in lung cancer management, with CDSS giving play to standardize lung cancer treatment protocols and facilitating multidisciplinary care. These systems efficiently integrate patient information with evidence-based guidelines, assisting teams in re-evaluating research findings when they are uncertain or inconsistent, considering relevant objective data, and making final decisions (Brown et al. 2022). Existing studies have demonstrated that CDSS, whether according to evidence (Pluyter et al. 2020) or employing rule and probabilistic reasoning (Sesen et al. 2014), can aid physicians in making better-quality decisions in lung cancer MDT. The strength of evidence-based CDSS is reflected through its capacity to offer a thorough understanding of factors affecting treatment decisions based on literature and guidelines, even when patient data remains unavailable, a fact supported by research (Sesen et al. 2014). However, CDSS which operates independently of other systems often necessitates extra effort and can disrupt clinicians’ workflows, thereby increasing the cognitive load on healthcare personnel (Sukums et al. 2015). To resolve this challenge, our study integrated evidence-based CDSS into the AI-MDT platform, whereby alignment with patient data from physician-confirmed imaging diagnoses and structured medical records offers accurate, personalized, and comprehensive medical recommendations under primary and confirmation. Based on physician-confirmed imaging diagnoses and structured medical record information, the AI-MDT platform retrieves and matches evidence-based knowledge base information to generate personalized diagnostic and treatment recommendations with traceable evidence. Following thorough discussion among at least 5 senior MDT specialists and final review by the primary specialist, the team formulates the diagnosis, treatment decisions, and follow-up plan.

In addition, AI technology extends extensively across detection (Lo et al. 2018; Sim et al. 2020), prediction of benign and malignant conditions (Ding et al. 2023; Zhou et al. 2023), and lesion information detection of lung lesions. Our algorithms integration achieves cross-scale feature fusion and multimodal detection segmentation of lung lesion images, enabling intelligent analysis and prediction of lung nodules and lymph nodes, including detection, localization, benign and malignant prediction, and TNM staging.

## Limitations

Whereas the AI-MDT platform demonstrates significant potential for improving efficiency in diagnosing and treating lung cancer, certain limitations exist. The platform maintains current confinement to internal use in our institution, thereby limiting its widespread application and impact across different medical settings. The addressing of this limitation will require future efforts to enhance the platform’s scalability and adaptability for broader dissemination and utilization. Regarding the second limitation, insufficient comparative analysis exists between the evidence-based recommendations of the CDSS and expert opinions, wherein the consistency between these two guiding approaches requires further validation to enhance the trustworthiness and reliability of the AI-MDT platform. Through addressing this issue, our engagement in continuous data collection evaluates the alignment of AI-MDT recommendations with traditional MDT decisions, while simultaneously evaluating patient satisfaction levels measures the platform’s effectiveness in improving clinical outcomes. Concerning the third limitation, while our strength is reflected by the adoption of advanced AI algorithms to establish evidence-based knowledge base and facilitate lesion diagnosis, the AI-MDT platform’s sustainability fundamentally depends on regular algorithm updates and timely integration of current evidence-based knowledge. Through ensuring the platform’s ongoing effectiveness, commitment toward integrating and updating more advanced algorithms maintains its technical edge and relevance in clinical practice. Lastly, with the development of high-throughput sequencing and parallel computing technologies, deep learning has been widely applied in genomics research, with advanced research utilizing parallel computing and deep learning techniques to process large-scale genetic data (Noor et al. 2025). The AI-MDT platform is a multi-modal data analysis platform but it does not yet incorporate comprehensive bioinformatics data, which could, which limits its diagnostic depth of lung cancer diagnosis and prognosis. Future work will focus on integrating bioinformatics datasets to enhance accuracy and personalized decision-making.

## Conclusion

In China, the increasing lung nodule detection rate generates increased diagnosis and treatment demands, necessitating intensive MDT member collaboration. This research addresses MDT diagnostic and treatment process challenges, specifically low collaboration efficiency and resource limitations, through development of an AI-assisted MDT platform. Through AI algorithm implementation, the AI-MDT platform executes multi-source heterogeneous data integration, whereby process automation achievement and evidence-based knowledge base construction occurs through LLM utilization, enabling real-time clinical practice decision support. Usability assessment findings demonstrate significant MDT efficiency enhancement through AI-MDT platform implementation, thereby illustrating broad AI application prospects in MDT collaboration while offering innovative tools and references for lung cancer diagnosis and treatment quality improvement.

## Data Availability

The datasets generated during the current study are not publicly available due to our institutional regulation, but are available from the corresponding author on reasonable request.
